# The relationship between testosterone replacement therapy and incidence of proximal humerus fractures in men: a matched retrospective analysis

**DOI:** 10.1016/j.xrrt.2025.100631

**Published:** 2025-12-01

**Authors:** Paul-Hugo Arcand, Simbarashe J. Peresuh, Joseph Confessore, Edward J. Testa, Matthew Quinn, Manjot Singh, Gabriella J. Avellino, Alan H. Daniels, Michel A. Arcand

**Affiliations:** a Department of Orthopaedic Surgery, The Warren Alpert Medical School of Brown University, Providence, RI, USA; b Department of Research, University Orthopedics Inc., Providence, RI, USA; c Department of Urology, The Warren Alpert Medical School of Brown University, Providence, RI, USA

**Keywords:** Testosterone, Testosterone replacement therapy, Hypogonadism, Proximal humerus fracture, Bone mineral density, Fracture risk

## Abstract

**Background:**

Anabolic androgenic steroid supraphysiologic dose use is linked to an increased risk of tendon rupture. Testosterone replacement therapy (TRT) is used to treat hypogonadism and is associated with increased bone mineral density. However, its relationship with fracture risk, particularly proximal humerus fractures (PHF), remains uncertain. This study evaluates the association between TRT and the incidence of PHFs using a large national database. We hypothesized that TRT use would be associated with a difference in the incidence of PHFs compared to a control group due to testosterone's role in maintaining bone mineral density.

**Methods:**

This retrospective study with one-to-one matching queried the PearlDiver Mariner165 dataset to obtain a random sample of 500,000 patients aged 35 to 75 who received TRT continuously for at least 3 months and a random control sample of 500,000 different patients. Of the targeted 1,000,000 patients, 335,753 pairs of patients were matched based on age, tobacco use, diabetes history, and the Charlson Comorbidity Index. Incidence of PHF was assessed over a 2-year follow-up using the International Classification of Diseases codes, with comparisons made across the different cohorts. Multivariable logistic regression was conducted to identify the association between TRT use with PHF in men. An alpha level of 0.01 was prespecified to reduce the risk of type I error and ensure statistical rigor.

**Results:**

In this matched-cohort study of 335,753 paired TRT and control patients, the mean age was 53.82 ± 10.58 years. Patients administered TRT demonstrated a higher incidence of PHF compared to the control cohort (0.029% vs. 0.005%, *P* < .001). Logistic regression analysis demonstrated that TRT was associated with a significantly increased risk of PHF (adjusted odds ratio 3.14; 95% confidence interval: 1.84-5.59; *P* < .001).

**Conclusions:**

In this retrospective matched-cohort study, TRT patients demonstrated an increase in 2-year incidence rates of PHF in men. These findings underscore the need for tailored patient management and provide actionable insights for orthopedic practice. Future studies of prospective design are needed to better address confounding factors, establish causation, and evaluate surgical outcomes in TRT patients.

Testosterone is an androgen, or male sex hormone, that plays a vital role in sexual development, bone metabolism, muscle growth, fat distribution, and libido. However, with increasing age, there is a natural decline in testosterone production, which significantly impacts bone mineral density (BMD) and other physiological functions.^22^ Proximal humerus fractures (PHF) account for about 6% of all adult fractures and 10% of all fractures in the age group 65 or older, making them the second most common fracture of the upper extremity.^2^^,^^10^ Geriatric PHF most often results from the combination of poor BMD and the increased propensity for ground-level falls that have been associated with advanced age.^2^ Previously established risk factors for PHF include low dietary calcium intake, diabetes mellitus, low physical activity, and the number of fractures since age 45.^1^

Hypogonadism has been shown to reduce BMD, thereby increasing the incidence of fractures.^26^ Testosterone replacement therapy (TRT), used to treat symptomatic hypogonadism, has been shown in some studies to increase BMD, particularly in hypogonadal men with osteoporosis and osteopenia.^26^ However, a few studies have failed to demonstrate this effect.^26^ Although Snyder et al found that TRT increased volumetric BMD and strength in the spine, this did not translate to fewer fragility fractures.^28^,^29^ In contrast to claims-based data, the recent The New England Journal of Medicine randomized trial by Snyder et al enrolled men with confirmed low testosterone, titrated transdermal TRT to mid-normal serum concentrations, measured levels serially, and monitored adherence by bottle-weighing; despite this rigorous protocol, clinical fractures were more frequent in the TRT arm (hazard ratio 1.43, 95% confidence interval (CI) 1.04-1.97). In that trial, randomization balanced baseline skeletal risk, such as osteoporosis, while bone density and falls were not measured during follow-up, and physical activity was not assessed.^27^ Snyder examined 3 categories of fractures: all fractures excluding the sternum, fingers, toes, facial bones, and skull; hip fractures; clinical vertebral fractures; and major osteoporotic fractures (hip, wrist, humerus, and spine fractures).^27^ In a database study, baseline skeletal risk must be addressed analytically. We therefore used matching and multivariable adjustment, including osteoporosis as a covariate, to examine the association between TRT exposure and 2-year PHF incidence in men. The purpose of this study is to further investigate the association between TRT and PHF in men. We hypothesized that TRT use would be associated with a difference in the incidence of PHFs compared to a control group, due to testosterone's role in maintaining BMD.

## Methods

### Study design

This study is a retrospective review of publicly available national data on the PearlDiver Mariner165 dataset (PearlDiver Technologies Inc., Colorado Springs, CO, USA). This dataset includes records for over 165 million United States patients covered by Medicare, Medicaid, government insurance, self-pay, and commercial insurance. The Mariner dataset spans from 2010 to the first quarter of 2022. All patients from 2010 to 2020 who met the inclusion criteria were considered. Additionally, these patients needed at least 2 years of follow-up data. Patient diagnoses were retrieved using the International Classification of Diseases (ICD) Ninth Revision prior to October 2015, and the Tenth Revision (ICD-10) thereafter.

### Study population

**Figure 1:**
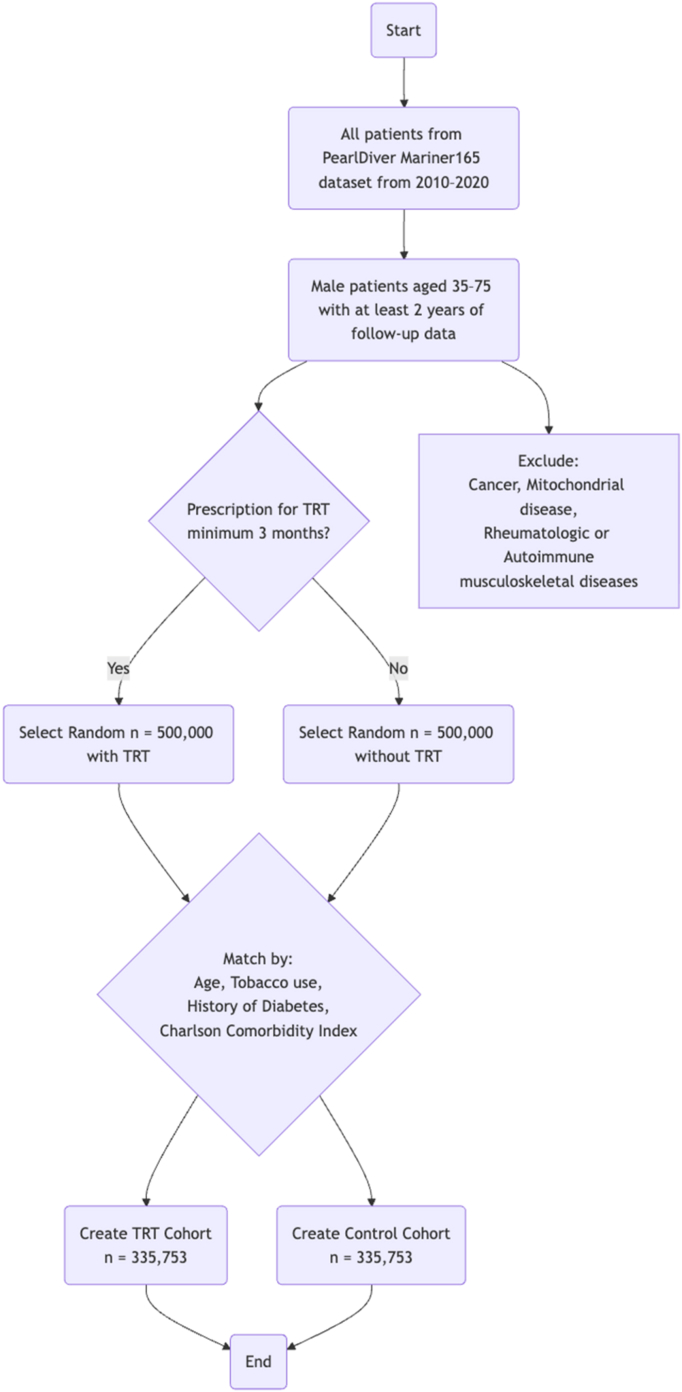
Flow diagram depicting patient selection, inclusion, and exclusion criteria for the analysis of incidence of proximal humerus fractures. *TRT*, testosterone replacement therapy.

Patients aged 35 to 75 with at least 2 years of follow-up data and a prescription for TRT for a minimum of 3 consecutive months were identified in the dataset from 2010 to 2022. Given the 2-year follow-up required, the last incidence of fracture was in 2020. The database does not include laboratory values (testosterone, sex hormone–binding globulin), dose, route, or adherence. No proxy for cumulative duration or compliance was available. For TRT patients, the index date was the earliest date on which 3 consecutive months of prescriptions had accrued; each matched control inherited the exposed patient's index date. Both groups required 2 years of follow-up after the index date. A randomly generated control group of patients within the same age range and with the same level of follow-up data, but without any TRT prescription, was also identified. This exposure definition (≥3 consecutive months of filled TRT prescriptions) and matched claims-based framework mirror prior large-scale work on TRT and fracture risk, including a matched analysis of hip fractures, thereby enabling comparability across skeletal sites while reflecting real-world prescribing patterns.^23^ Patients with cancer, mitochondrial disease, rheumatologic musculoskeletal disease, or autoimmune musculoskeletal disease were excluded from the study. We sought to match 500,000 patients with TRT and 500,000 without TRT randomly to create the TRT and control groups. Of these 1,000,000 patients, 335,753 pairs of patients were matched based on age, tobacco use, diabetes history, and the Charlson Comorbidity Index (CCI) (Fig. 1). We did not restrict inclusion to patients with a coded diagnosis of hypogonadism because diagnosis coding is inconsistently applied in administrative data and is not linked to laboratory confirmation. Restricting to coded hypogonadism would risk misclassification, reduce sample size, and limit external validity. Our design was aimed at capturing the association between prescription-defined TRT exposure and fracture outcomes.

### Data extraction

Patient demographics, including sex, age, CCI, diabetes, and tobacco use, were extracted from the dataset. The incidence of PHF over the 2-year follow-up period was identified using ICD 9 and 10 diagnostic codes. All PHFs were included in the study (Supplementary Appendix S1).

### Statistical analysis

The incidence of PHF was calculated for all cohorts, and a comparison was made using the exact Poisson method. Two-sided Chi-square tests facilitated bivariate comparisons between the 2 matched groups, and *t*-tests were used to compare the ages of the groups. To compare the 2 cohorts while accounting for other specific comorbidities such as hypogonadism, chronic kidney disease, osteoporosis, overweight/obesity (body mass index >25), class III obesity (body mass index >40), alcohol use, osteoarthritis, lung disease, congestive heart failure, and dementia, multivariable logistic regression was employed. These variables were included in the regression model to control for factors that might influence the initial injury rate.^1^^,^^23^ Two analyses were performed, the first was an unmatched analysis conducted on the full cohort of 500,000 patients in each group (Table I). The second was a matched analysis conducted on the 335,753 matched pairs (Table I). Adjusted odds ratios (*a*ORs) and their 95% CIs were generated and reported for each comparison (Table II). Given the potential for multiple comparisons, an alpha level of 0.01 was prespecified to reduce the risk of type I error and ensure statistical rigor. All statistical analyses were conducted using the R Statistical Package (R Core Team 2022 v4.2.1; R Foundation for Statistical Computing, Vienna, Austria) embedded within PearlDiver.

**Table I:** Demographic and baseline characteristics of the nonmatched cohorts (500,000 pairs) and matched cohorts (335,753 pairs).

	Nonmatched			Matched		
	TRT	Control	*P* value	TRT	Control	*P* value
Count, n	500,000	500,000		335,753	335,753	
Age, mean (yrs) (±SD)	55.12 (10.77)	55.91 (11.44)	**<.001**	53.82 (10.58)	53.82 (10.58)	>.999
CCI, mean (±SD)	0.82 (1.46)	0.12 (0.45)	**<.001**	0.17 (0.54)	0.17 (0.54)	>.999
Tobacco Use, n (%)	175,219 (35.04)	188,564 (37.71)	**<.001**	106.497 (31.71)	106.497 (31.71)	>.999
Diabetes, n (%)	189,978 (37.99)	183,614 (36.72)	**<.001**	100,306 (29.87)	100,306 (29.87)	>.999

*CCI*, Charlson Comorbidity Index; *SD*, standard deviation; *TRT*, testosterone replacement therapy.

Bold indicates statistical significance (*P* < .01).

**Table II:** Incidence of proximal humerus fractures over 2 years for the matched TRT cohort (n = 335,753) and matched control cohort (n = 335,753).

Cohort	Count, n	TRT, n (%)	Control, n (%)	*P* value	*a*OR (95% CI)	*P* value
Total	335,753	97 (0.030)	16 (0.005)	**<.001**	3.14 (1.84-5.59)	**<.001**
35-45 yr	72,776	20 (0.028)	1 (0.001)	**<.001**	4.93 (1.17-33.56)	.049
46-55 yr	103,955	32 (0.031)	2 (0.002)	**<.001**	7.52 (2.47-32.73)	**.002**
56-65 yr	93,666	27 (0.029)	9 (0.010)	.034	2.06 (0.80-5.94)	.150
66-75 yr	65,356	18 (0.028)	4 (0.006)	.198	1.27 (0.51-3.46)	.618

*aOR*, adjusted odds ratio; *CI*, confidence interval; *TRT*, testosterone replacement therapy.

Bold indicates statistical significance (*P* < .01). *a*OR adjusted using multivariable logistic regression to additionally control for hypogonadism, chronic kidney disease, osteoporosis, overweight/obesity (body mass index >25), class III obesity (body mass index >40), alcohol use, osteoarthritis, lung disease, congestive heart failure, and dementia.

## Results

### Patient characteristics

Initially, 500,000 patients were targeted and identified in each cohort (TRT and control), but after applying exclusion criteria and one-to-one matching, 335,753 patients remained in each cohort (TRT and control) for the final multivariable analysis (Fig. 1). Before matching, both groups had statistically significant demographic differences in age, CCI, male sex, tobacco, and diabetes (Table I). The control group had a higher mean age (55.91 ± 11.44 years) than the TRT group (55.12 ± 10.77 years). On the other hand, the TRT group had a higher mean CCI (0.82 ± 1.46 vs. 0.12 ± 0.45), higher proportions of diabetes (37.99% vs. 36.72%), and lower tobacco use (35.04% vs. 37.71%). After the exclusion criteria and 1:1 exact matching process were met, 335,753 subjects comprised the TRT and control groups. Following the matching process, the differences no longer persisted. These patients had an average age of 53.82 (±10.58) years and an average CCI of 0.17 (±0.54). Of this matched cohort, 31.71% were tobacco users, and 29.87% were diabetic (Table I). Table II depicts the number of patients in each of the age-specific cohorts.

### Proximal humerus fracture associated risk factors

Over the 2-year follow-up period, the incidence of PHF was 28.9 per 100,000 person-years for the TRT group, compared to 4.8 per 100,000 person-years for the control group (*P* < .001). The TRT group exhibited a higher incidence of PHF compared to the control group (0.029% vs. 0.005%, *P* < .001) (Table II). When stratified by age, this difference was significant in the 35-45 age group (0.028% vs. 0.001%, *P* < .001) and the 46-55 age group (0.031% vs. 0.002%, *P* < .001). The adjusted multivariable regression showed that patients in the TRT group were significantly more likely to experience a PHF than patients in the control group (*a*OR = 3.14, 95% CI, 1.84-5.59, *P* < .001). This was also seen after age stratification, as reported by the 46-55 age group, the TRT cohort had a significantly increased risk of PHF (*a*OR = 7.52; 95% CI: 2.47-32.73; *P* = .002).

## Discussion

TRT has gained widespread clinical use to treat hypogonadism and age-related testosterone decline, improving BMD, muscle mass, and overall physical performance. However, evidence from our study and existing literature challenges the presumption that TRT reduces fracture risk. Our analysis of the PearlDiver Mariner165 dataset revealed a significantly higher incidence of PHFs in TRT users compared to matched controls. After matching the control and TRT groups, the TRT group was 3.1-fold higher odds to experience PHF than the control group.

While exogenous testosterone use is classically associated with anabolic steroids aimed at improving athletic performance, it is commonly used therapeutically for conditions resulting in testosterone deficiency, such as hypogonadism.^16^^,^^33^ When administered for restoration of physiologic levels, the administration of exogenous testosterone is referred to as TRT. In recent years, there has been an increasing number of individuals receiving TRT, particularly men between the ages of 18 and 45 years.^24^ Although TRT has been shown to increase BMD, its effect on fracture incidence remains uncertain, as there is a paucity of available literature on the topic. Our retrospective study leveraged the PearlDiver Mariner165 database, encompassing over 165 million patient records. We examined patients aged 35 to 75 who had been prescribed TRT for at least three consecutive months and had at least two years of follow-up. By using exact matching for age, sex, tobacco use, diabetes, and the CCI, we created comparable TRT and control groups, minimizing confounding variables. In contrast, Snyder et al conducted a large randomized controlled trial that showed TRT was associated with a higher incidence of clinical fractures in hypogonadal men (hazard ratio, 1.43; 95% CI, 1.04-1.97).^27^ Although randomized controlled trials are the gold standard for establishing causality, Snyder's study did not match participants for critical risk factors. Consistent with Snyder et al, we note that physical activity and risk-taking behavior were not assessed; thus, behavioral mediators were not evaluated in either dataset, even though randomization should balance baseline behaviors in the trial. Our analysis complements those trial data by estimating real-world associations absent laboratory confirmation or standardized dosing, while adjusting analytically for coded osteoporosis and other comorbidities, providing evidence of the association between TRT and PHF. While observational and results should be interpreted as population-level associations, our study offers a more representative cross-section of the general population, enhancing external validity. Though the Snyder study did include the humerus, it did not test a matched population or account for other risk factors associated with fragility factors. Furthermore, the U-shaped association between testosterone levels and fracture risk demonstrated by Yeap et al adds depth to our findings, indicating that both low and excessively high testosterone levels can elevate fracture risk.^34^ This observation highlights the complex and nonlinear effects of testosterone on bone health. This nuanced relationship underscores the importance of studying TRT in real-world settings, as supraphysiological testosterone levels achieved through supplementation may contribute to fracture susceptibility. Our study's findings align with this evidence, reinforcing the complexity of TRT's impact on skeletal integrity.

Though TRT improves BMD, bone strength is not solely determined by density.^29^ Factors such as microarchitecture, geometry, and cortical thickness are critical to overall bone integrity. Filho Marquardt and Da Ros confirmed that TRT enhances BMD in hypogonadal men but did not establish a direct reduction in fracture incidence.^20^ This suggests that TRT may increase bone density without adequately improving bone quality, potentially leaving bones more brittle and fracture prone. Based on the theory of TRT providing an increase in BMD, one could intuitively hypothesize that TRT could potentially decrease the risk of fragility fractures, such as PHFs.^27^ Bone metabolism is influenced by numerous endogenous hormones including vitamin D, estrogen, and testosterone.^3^^,^^8^^,^^9^^,^^14^^,^^23^^,^^31^ Testosterone can promote bone formation through both direct and indirect mechanisms. It directly enhances osteoblast activation, increases androgen receptor expression in osteoblasts, and increases differentiation in osteoblasts and chondrocytes.^26^ Testosterone's indirect effects on bone metabolism are mediated via the regulation of cytokines and growth factors, such as interleukin-6, which is downregulated by testosterone and, therefore, inhibits osteoclasts.^26^ TRT has been associated with shoulder pathology. Prior to considering its association with PHF, TRT has been associated with an increase in the risk of rotator cuff tears.^32^ Multiple prior investigations have found that BMD is positively correlated with serum testosterone levels in men.^14^^,^^15^^,^^26^^,^^30^ For example, exogenous testosterone administration has been shown to improve not only BMD but also libido, body composition, lean muscle mass, depressive symptoms, and cognition, while androgen deprivation therapy for prostate cancer typically results in decreased BMD and increased osteoporosis.^4^, ^5^, ^6^, ^7^^,^^11^, ^12^, ^13^, ^14^^,^^17^, ^18^, ^19^^,^^21^^,^^25^, ^26^, ^27^^,^^35^

Additionally, many individuals prescribed TRT have a history of hypogonadism, leading to long-term BMD deficits that TRT may not fully reverse. Snyder et al highlighted that patients with preexisting osteoporosis may remain vulnerable to fractures despite TRT-related BMD improvements.^27^ This background risk is exacerbated by TRT-induced behavioral and physiological changes, collectively increasing fracture risk.

The mechanisms underlying the observed paradox of increased BMD but higher fracture incidence in TRT users remain incompletely understood and warrant further investigation in prospective cohorts with laboratory confirmation and behavioral assessment. Our study shows that TRT exposure is associated with increased PHF risk in men, complementing high-level evidence that TRT improves BMD yet paradoxically raises fracture incidence.^27^ Population data also describe a U-shaped relationship between testosterone and fracture risk.^34^ Although the absolute incidence of PHF among men prescribed TRT was low, in this landscape, identifying risk factors that inform prevention and counseling is increasingly important. Together, these data suggest that TRT exposure should be recognized not as a determinant of fracture management, but as a clinical risk signal. For the orthopedic surgeon, practical implications include documenting TRT use as part of the patient history, considering fragility evaluation and secondary prevention strategies such as BMD testing and vitamin D/calcium optimization, and engaging primary care, urology, or endocrinology colleagues in shared care. For patients sustaining PHF while on TRT, counseling should incorporate recognition of systemic and activity-related risk factors, while operative versus nonoperative management decisions continue to follow established criteria based on age, comorbidities, and fracture morphology.

### Limitations and future research directions

Several limitations affect this study. Firstly, the retrospective and observational nature of the study limits causal inference between TRT use and PHF. While this study does use one-to-one matching on patient demographics and the CCI and then uses a multivariable regression analysis to control for several confounding medical conditions, several other factors place patients at increased risk of fractures that could not be captured in this study, such as patients' activity levels at baseline and fall risk. Secondly, the study relies on the consistent and accurate use of ICD-10 codes and TRT prescription records. This dependence on database accuracy is crucial for forming the desired cohorts. The study does not account for the reasons for TRT initiation, the dosing regimen and compliance, formulations (injectable vs. transdermal) were not differentiated, or laboratory data (serum testosterone, sex hormone-binding globulin) at baseline and over the study period. Results should therefore be interpreted as population-level associations. Exposure was defined by prescription records, and no proxy measure for long-term compliance or cumulative treatment duration could be applied. Although osteoporosis was included as a covariate, administrative data cannot capture disease severity, BMD, or nonosteoporotic metabolic bone disease, leaving residual confounding possible. Unlike randomized designs, which balance baseline risk through randomization, database studies remain subject to unmeasured confounding. A comparator group of hypogonadal men untreated with TRT would not be constructed reliably because hypogonadism is inconsistently coded and not linked to laboratory confirmation in claims data; restricting to coded hypogonadism would likely introduce misclassification and reduce external validity.

Future research investigating these variables in conjunction with TRT use is warranted to better understand the role of TRT in PHF. Future prospective cohort studies should explore TRT's long-term effects on fracture risk and the impact of behavioral factors on fracture risk in TRT patients. In addition, investigations on the dose-response relationships between TRT and bone quality could help orthopedic surgeons examine the role of adjunctive therapies (osteoporosis medications) in mitigating fracture risk among TRT users.

## Conclusion

In this retrospective matched-cohort study, TRT was associated with an increased 2-year incidence rate of PHF. When stratified by age, this finding was significant for middle-aged and elderly men. These findings support risk awareness and secondary prevention but do not change operative indications and procedures. Previous bodies of work have investigated the benefits of testosterone on BMD; however, there is still limited evidence supporting the notion that TRT can prevent bone fracture incidence. Future research should focus on understanding the intricate relationships between TRT, bone integrity, and fracture risk, ultimately guiding evidence-based clinical practices to safeguard patient health.

## Disclaimers:

Funding: No funding was disclosed by the authors.

Conflicts of interest: Alan H. Daniels reports being a paid consultant for EOS, Orthofix, Inc., SpineArt, Medtronic/Medicrea; Stryker; research support from Orthofix, Inc.; and publishing royalties and financial or material support from Springer all outside submitted work. The other authors, their immediate families, and any research foundation with which they are affiliated have not received any financial payments or other benefits from any commercial entity related to the subject of this article.

## References

[1] Albright J.A., Lou M., Rebello E., Ge J., Testa E., Daniels A. (2023). Testosterone replacement therapy is associated with increased odds of Achilles tendon injury and subsequent surgery: a matched retrospective analysis. J Foot Ankle Res.

[2] Alrabaa R.G., Ma G., Truong N.M., Lansdown D., Feeley B., Zhang A. (2022). Trends in surgical treatment of proximal humeral fractures and analysis of postoperative complications over a decade in 384,158 patients. JB JS Open Access.

[3] Bagatell C.J., Heiman J.R., Matsumoto A.M., Rivier J.E., Bremner W.J. (1994). Metabolic and behavioral effects of high-dose, exogenous testosterone in healthy men. J Clin Endocrinol Metab.

[4] Baker H.P., Gutbrod J., Strelzow J.A., Maassen N.H., Shi L. (2022). Management of proximal humerus fractures in adults—a scoping review. J Clin Med.

[5] Beks R.B., Ochen Y., Frima H., Smeeing D., Meijden O., Timmers T. (2018). Operative versus nonoperative treatment of proximal humeral fractures: a systematic review, meta-analysis, and comparison of observational studies and randomized controlled trials. J Shoulder Elbow Surg.

[6] Bolam S.M., Wells Z., Tay M.L., Frampton C.M.A., Coleman B., Dalgleish A. (2024). Reverse total shoulder arthroplasty for acute proximal humeral fracture has comparable 10-year outcomes to elective indications: results from the New Zealand Joint Registry. J Shoulder Elbow Surg.

[7] Boloña E.R., Uraga M.V., Haddad R.M., Tracz M., Sideras K., Kennedy C. (2007). Testosterone use in men with sexual dysfunction: a systematic review and meta-analysis of randomized placebo-controlled trials. Mayo Clin Proc.

[8] Bowling G.C., Albright J.A., Maloney T.J., Quinn M.S., Daniels A.H., Chesnut G.T. (2024). Poor bone mineral density is associated with increased risk of urological bone metastases. Urology.

[9] Chang K., Albright A., Quinn M., Khatri S., Zhao L., Byrne R. (2023). A diagnosis of vitamin D deficiency is associated with increased rates of primary patellar instability and need for recurrent surgical stabilization. Orthop J Sports Med.

[10] Chu S.P., Kelsey J.L., Keegan T.H.M., Sternfeld B., Prill M., Quesenberry C. (2004). Risk factors for proximal humerus fracture. Am J Epidemiol.

[11] Hageman M.G.J.S., Meijer D., Stufkens S.A., Ring D., Doornberg J.N., Steller E.P. (2016). Proximal humeral fractures: nonoperative versus operative treatment. Arch Trauma Res.

[12] Handoll H., Brealey S., Rangan A., Keding A., Corbacho B., Jefferson L. (2015). The ProFHER (PROximal Fracture of the Humerus: evaluation by Randomisation) trial—a pragmatic multicentre randomised controlled trial evaluating the clinical effectiveness and cost-effectiveness of surgical compared with non-surgical treatment for proximal fracture of the humerus in adults. Health Technol Assess.

[13] Handoll H.H., Elliott J., Thillemann T.M., Aluko P., Brorson S. (2022). Interventions for treating proximal humeral fractures in adults. Cochrane Database Syst Rev.

[14] Isidori A.M., Giannetta E., Greco E.A., Gianfrilli D., Bonifacio V., Isidori A. (2005). Effects of testosterone on body composition, bone metabolism and serum lipid profile in middle-aged men: a meta-analysis. Clin Endocrinol (Oxf).

[15] Jones I.A., Togashi R., Hatch G.F.R., Weber A.E., Vangsness C.T. (2018). Anabolic steroids and tendons: a review of their mechanical, structural, and biologic effects. J Orthop Res.

[16] Kanayama G., Hudson J.I., Pope H.G. (2008). Long-term psychiatric and medical consequences of anabolic–androgenic steroid abuse: a looming public health concern?. Drug Alcohol Depend.

[17] Kenny A.M., Bellantonio S., Gruman C.A., Acosta R.D., Prestwood K.M. (2002). Effects of transdermal testosterone on cognitive function and health perception in older men with low bioavailable testosterone levels. J Gerontol A Biol Sci Med Sci.

[18] Lapner P., Sheth U., Nam D., Schemitsch E., Guy P., Robin R. (2023). Management of proximal humeral fractures in adults: a systematic review and meta-analysis. J Orthop Trauma.

[19] Launonen A.P., Sumrein B.O., Reito A., Lepola V., Paloneva J., Berg H. (2023). Surgery with locking plate or hemiarthroplasty versus nonoperative treatment of 3-4-part proximal humerus fractures in older patients (NITEP): an open-label randomized trial. PLoS Med.

[20] Marquardt N., Ros C.T.D. (2023). Can we recommend varicocele surgery for men with hypogonadism?. Int Braz J Urol.

[21] Matheron G., Stringfellow T., Lebe M., Domos P. (2025). Conserve or reverse? Outcomes of conservative treatment versus reverse shoulder arthroplasty in displaced 3- and 4-part proximal humeral fractures in patients over 60 years. J Shoulder Elbow Surg.

[22] Nassar G.N., Leslie S.W. (2025). StatPearls.

[23] Peresuh S.J., Arcand P.H., Confessore J., Parvaresh-Rizi A., Testa E.J., Quinn M. (2025). A matched retrospective analysis: the relationship between testosterone replacement therapy and the incidence of hip fractures. J Am Acad Orthop Surg.

[24] Rao P.K., Boulet S.L., Mehta A., Hotaling J., Eisenberg M., Honig S. (2017). Trends in testosterone replacement therapy use from 2003 to 2013 among reproductive-age men in the United States. J Urol.

[25] Sebastiá-Forcada E., Cebrián-Gómez R., Lizaur-Utrilla A., Gil-Guillén V. (2014). Reverse shoulder arthroplasty versus hemiarthroplasty for acute proximal humeral fractures: a blinded, randomized, controlled, prospective study. J Shoulder Elbow Surg.

[26] Shigehara K., Izumi K., Kadono Y., Mizokami A. (2021). Testosterone and bone health in men: a narrative review. J Clin Med.

[27] Sinha-Hikim I., Roth S.M., Lee M.I., Bhasin S. (2003). Testosterone-induced muscle hypertrophy is associated with an increase in satellite cell number in healthy, young men. Am J Physiol Endocrinol Metab.

[28] Snyder P.J., Bauer D.C., Ellenberg S.S., Cauley J., Buhr K., Bhasin S. (2024). Testosterone treatment and fractures in men with hypogonadism. N Engl J Med.

[29] Snyder P.J., Kopperdahl D.L., Stephens-Shields A.J., Ellenberg S., Cauley J., Ensrud K. (2017). Effect of testosterone treatment on volumetric bone density and strength in older men with low testosterone: a controlled clinical trial. JAMA Intern Med.

[30] Straftis A.A., Gray P.B. (2019). Sex, energy, well-being and low testosterone: an exploratory survey of U.S. men’s experiences on prescription testosterone. Int J Environ Res Public Health.

[31] Streicher C., Heyny A., Andrukhova O., Haigl B., Slavic S., Schüler C. (2017). Estrogen regulates bone turnover by targeting RANKL expression in bone lining cells. Sci Rep.

[32] Testa E.J., Albright J.A., Hartnett D., Lemme N., Daniels A., Owens B. (2023). The relationship between testosterone therapy and rotator cuff tears, repairs, and revision repairs. J Am Acad Orthop Surg.

[33] Weber A.E., Gallo M.C., Bolia I.K., Cleary E.J., Schroeder T.E., Hatch G.F. (2022). Anabolic androgenic steroids in orthopaedic surgery: current concepts and clinical applications. J Am Acad Orthop Surg Glob Res Rev.

[34] Yeap B.B., Alfonso H., Chubb S.A.P., Center J., Beilin J., Hankey G. (2020). U-shaped association of plasma testosterone, and no association of plasma estradiol, with incidence of fractures in men. J Clin Endocrinol Metab.

[35] Zitzmann M. (2020). Testosterone, mood, behaviour and quality of life. Andrology.

