# Supplementary material for: The relationship between testosterone replacement therapy and incidence of proximal humerus fractures in men: a matched retrospective analysis
Source: JSES Rev Rep Tech. 2025 Dec 1;6(2):100631. doi: 10.1016/j.xrrt.2025.100631 (PMC12810334; doi:10.1016/j.xrrt.2025.100631)
Supplement: Supplementary Appendix S1 [file mmc1.docx]

| ICD-9 Codes for Proximal Humerus Fractures | |
| --- | --- |
| Codes | Description |
| 81200 | Closed fracture of unspecified part of upper end of humerus |
| 81210 | Open fracture of unspecified part of upper end of humerus |
| 81201 | Closed fracture of surgical neck of humerus |
| 81211 | Open fracture of surgical neck of humerus |
| 81203 | Closed fracture of greater tuberosity of humerus |
| 81213 | Open fracture of greater tuberosity of humerus |
| 81209 | Other closed fracture of upper end of humerus |
| 81219 | Other open fracture of upper end of humerus |
| 81202 | Closed fracture of anatomical neck of humerus |
| 81212 | Open fracture of anatomical neck of humerus |
| ICD-10 Codes for Proximal Humerus Fractures | |
| Codes | Description |
| S42201A | Unspecified fracture of upper end of right humerus, initial encounter for closed fracture |
| S42201B | Unspecified fracture of upper end of right humerus, initial encounter for open fracture |
| S42202A | Unspecified fracture of upper end of left humerus, initial encounter for closed fracture |
| S42202B | Unspecified fracture of upper end of left humerus, initial encounter for open fracture |
| S42209A | Unspecified fracture of upper end of unspecified humerus, initial encounter for closed fracture |
| S42211A | Unspecified displaced fracture of surgical neck of right humerus, initial encounter for closed fracture |
| S42211B | Unspecified displaced fracture of surgical neck of right humerus, initial encounter for open fracture |
| S42212A | Unspecified displaced fracture of surgical neck of left humerus, initial encounter for closed fracture |
| S42212B | Unspecified displaced fracture of surgical neck of left humerus, initial encounter for open fracture |
| S42213A | Unspecified displaced fracture of surgical neck of unspecified humerus, initial encounter for closed fracture |
| S42213B | Unspecified displaced fracture of surgical neck of unspecified humerus, initial encounter for open fracture |
| S42214A | Unspecified nondisplaced fracture of surgical neck of right humerus, initial encounter for closed fracture |
| S42214B | Unspecified nondisplaced fracture of surgical neck of right humerus, initial encounter for open fracture |
| S42215A | Unspecified nondisplaced fracture of surgical neck of left humerus, initial encounter for closed fracture |
| S42215B | Unspecified nondisplaced fracture of surgical neck of left humerus, initial encounter for open fracture |
| S42216A | Unspecified nondisplaced fracture of surgical neck of unspecified humerus, initial encounter for closed fracture |
| S42216B | Unspecified nondisplaced fracture of surgical neck of unspecified humerus, initial encounter for open fracture |
| S42221A | 2-part displaced fracture of surgical neck of right humerus, initial encounter for closed fracture |
| S42221B | 2-part displaced fracture of surgical neck of right humerus, initial encounter for open fracture |
| S42222A | 2-part displaced fracture of surgical neck of left humerus, initial encounter for closed fracture |
| S42222B | 2-part displaced fracture of surgical neck of left humerus, initial encounter for open fracture |
| S42223A | 2-part displaced fracture of surgical neck of unspecified humerus, initial encounter for closed fracture |
| S42223B | 2-part displaced fracture of surgical neck of unspecified humerus, initial encounter for open fracture |
| S42224A | 2-part nondisplaced fracture of surgical neck of right humerus, initial encounter for closed fracture |
| S42224B | 2-part nondisplaced fracture of surgical neck of right humerus, initial encounter for open fracture |
| S42225A | 2-part nondisplaced fracture of surgical neck of left humerus, initial encounter for closed fracture |
| S42225B | 2-part nondisplaced fracture of surgical neck of left humerus, initial encounter for open fracture |
| S42226A | 2-part nondisplaced fracture of surgical neck of unspecified humerus, initial encounter for closed fracture |
| S42226B | 2-part nondisplaced fracture of surgical neck of unspecified humerus, initial encounter for open fracture |
| S42231A | 3-part fracture of surgical neck of right humerus, initial encounter for closed fracture |
| S42231B | 3-part fracture of surgical neck of right humerus, initial encounter for open fracture |
| S42232A | 3-part fracture of surgical neck of left humerus, initial encounter for closed fracture |
| S42232B | 3-part fracture of surgical neck of left humerus, initial encounter for open fracture |
| S42239A | 3-part fracture of surgical neck of unspecified humerus, initial encounter for closed fracture |
| S42239B | 3-part fracture of surgical neck of unspecified humerus, initial encounter for open fracture |
| S42241A | 4-part fracture of surgical neck of right humerus, initial encounter for closed fracture |
| S42241B | 4-part fracture of surgical neck of right humerus, initial encounter for open fracture |
| S42242A | 4-part fracture of surgical neck of left humerus, initial encounter for closed fracture |
| S42242B | 4-part fracture of surgical neck of left humerus, initial encounter for open fracture |
| S42249A | 4-part fracture of surgical neck of unspecified humerus, initial encounter for closed fracture |
| S42249B | 4-part fracture of surgical neck of unspecified humerus, initial encounter for open fracture |
| S42251A | Displaced fracture of greater tuberosity of right humerus, initial encounter for closed fracture |
| S42251B | Displaced fracture of greater tuberosity of right humerus, initial encounter for open fracture |
| S42252A | Displaced fracture of greater tuberosity of left humerus, initial encounter for closed fracture |
| S42252B | Displaced fracture of greater tuberosity of left humerus, initial encounter for open fracture |
| S42253A | Displaced fracture of greater tuberosity of unspecified humerus, initial encounter for closed fracture |
| S42253B | Displaced fracture of greater tuberosity of unspecified humerus, initial encounter for open fracture |
| S42254A | Nondisplaced fracture of greater tuberosity of right humerus, initial encounter for closed fracture |
| S42254B | Nondisplaced fracture of greater tuberosity of right humerus, initial encounter for open fracture |
| S42255A | Nondisplaced fracture of greater tuberosity of left humerus, initial encounter for closed fracture |
| S42255B | Nondisplaced fracture of greater tuberosity of left humerus, initial encounter for open fracture |
| S42256A | Nondisplaced fracture of greater tuberosity of unspecified humerus, initial encounter for closed fracture |
| S42256B | Nondisplaced fracture of greater tuberosity of unspecified humerus, initial encounter for open fracture |
| S42261A | Displaced fracture of lesser tuberosity of right humerus, initial encounter for closed fracture |
| S42261B | Displaced fracture of lesser tuberosity of right humerus, initial encounter for open fracture |
| S42262A | Displaced fracture of lesser tuberosity of left humerus, initial encounter for closed fracture |
| S42262B | Displaced fracture of lesser tuberosity of left humerus, initial encounter for open fracture |
| S42263A | Displaced fracture of lesser tuberosity of unspecified humerus, initial encounter for closed fracture |
| S42263B | Displaced fracture of lesser tuberosity of unspecified humerus, initial encounter for open fracture |
| S42264A | Nondisplaced fracture of lesser tuberosity of right humerus, initial encounter for closed fracture |
| S42264B | Nondisplaced fracture of lesser tuberosity of right humerus, initial encounter for open fracture |
| S42265A | Nondisplaced fracture of lesser tuberosity of left humerus, initial encounter for closed fracture |
| S42265B | Nondisplaced fracture of lesser tuberosity of left humerus, initial encounter for open fracture |
| S42266A | Nondisplaced fracture of lesser tuberosity of unspecified humerus, initial encounter for closed fracture |
| S42266B | Nondisplaced fracture of lesser tuberosity of unspecified humerus, initial encounter for open fracture |
| S42271A | Torus fracture of upper end of right humerus, initial encounter for closed fracture |
| S42272A | Torus fracture of upper end of left humerus, initial encounter for closed fracture |
| S42279A | Torus fracture of upper end of unspecified humerus, initial encounter for closed fracture |
| S42291A | Other displaced fracture of upper end of right humerus, initial encounter for closed fracture |
| S42291B | Other displaced fracture of upper end of right humerus, initial encounter for open fracture |
| S42292A | Other displaced fracture of upper end of left humerus, initial encounter for closed fracture |
| S42292B | Other displaced fracture of upper end of left humerus, initial encounter for open fracture |
| S42293A | Other displaced fracture of upper end of unspecified humerus, initial encounter for closed |
| S42293B | Other displaced fracture of upper end of unspecified humerus, initial encounter for open fracture |
| S42294A | Other nondisplaced fracture of upper end of right humerus, initial encounter for closed fracture |
| S42294B | Other nondisplaced fracture of upper end of right humerus, initial encounter for open fracture |
| S42295A | Other nondisplaced fracture of upper end of left humerus, initial encounter for closed fracture |
| S42295B | Other nondisplaced fracture of upper end of left humerus, initial encounter for open fracture |
| S42296A | Other nondisplaced fracture of upper end of unspecified humerus, initial encounter for closed fracture |
| S42296B | Other nondisplaced fracture of upper end of unspecified humerus, initial encounter for open fracture |
